# Hemoglobins F, A_2_, and E levels in Laotian children aged 6‐23 months with Hb E disorders: Effect of age, sex, and thalassemia types

**DOI:** 10.1111/ijlh.13164

**Published:** 2020-02-12

**Authors:** Benchawan Kingchaiyaphum, Kanokwan Sanchaisuriya, Goonnapa Fucharoen, Attawut Chaibunruang, Sonja Y. Hess, Guy‐Marino Hinnouho, Maxwell A. Barffour, Kimbery R. Wessells, Sengchanh Kounnavong, Supan Fucharoen

**Affiliations:** ^1^ Graduate School Khon Kaen University Khon Kaen Thailand; ^2^ Center for Research and Development of Medical Diagnostic Laboratories (CMDL) Faculty of Associated Medical Sciences Khon Kaen University Khon Kaen Thailand; ^3^ Institute for Global Nutrition University of California Davis CA USA; ^4^ Lao Tropical and Public Health Institute Ministry of Health Vientiane Lao

**Keywords:** hemoglobin A_2_, hemoglobin E, hemoglobin F, hemoglobinopathy, thalassemia, young children

## Abstract

**Introduction:**

Determination of hemoglobins (Hbs) F, A_2,_ and E is crucial for diagnosis of thalassemia. This study determined the levels of Hbs F, A_2,_ and E in children aged 6‐23 months and investigated the effect of age, sex, and types of thalassemia on the expression of these Hbs.

**Methods:**

A total of 698 blood samples of Laotian children including 272 non‐Hb E, 271 Hb E heterozygotes, and 155 Hb E homozygotes were collected. Hb profiles were determined using the capillary zone electrophoresis. Coinheritance of α‐thalassemia and the homozygosity for Hb E mutation were checked by PCR‐based assay.

**Results:**

Children heterozygous and homozygous for Hb E had significantly higher Hb F and A_2_ levels than non‐Hb E children (median Hb F = 1.1% for non‐Hb E group, 2.7% for Hb E heterozygotes, and 9.4% for Hb E homozygotes; median Hb A_2_ = 2.6% for non‐Hb E group, 3.8% for Hb E heterozygotes, and 5.2% for Hb E homozygotes). The median Hb E levels were 21.9% for Hb E heterozygotes and 85.3% for Hb E homozygotes. Comparing within group, there was a statistically significant difference between children with and without an α‐gene defect for Hb A_2_ and E, but not Hb F. Based on a multiple regression analysis, age and sex were significantly associated with the expression of Hb F and A_2_ but not Hb E.

**Conclusions:**

Our findings can guide the development of a diagnostic approach to thalassemia in children aged 6‐23 months.

## 1 INTRODUCTION

Hemoglobin F (Hb F; α_2_γ_2_) is a major hemoglobin during the fetal period. In neonates, Hb F normally declines gradually and reaches normal levels of less than 1% of total hemoglobin at the age of around 10 months.^1^ Meanwhile, adult hemoglobin A (Hb A; α_2_β_2_) increases gradually in replacement of Hb F. Small amounts of minor adult hemoglobin, hemoglobin A_2_ (Hb A_2_; α_2_δ_2_), can be detected after birth. Its expression increases gradually and has been shown to reach a normal adult level at age 5‐6 months.^2^ Inherited disorders of hemoglobin could result in varying levels of Hb F and A_2_ depending mainly on the types of the affected gene. In adults heterozygous for β‐thalassemia (β‐thal), the level of Hb A_2_ is usually higher than 3.5% with varying levels of Hb F. A persistently high Hb F level is found in patients with β‐thal as well as in those who carry high Hb F determinants.^3^, ^4^

Hemoglobin E (Hb E; α_2_^E^_2_), a structural Hb variant commonly found in South East Asia, results from a single nucleotide base substitution (G to A) at codon 26 of the β‐globin gene. The mutation activates a cryptic splice site in the mRNA and leads to a reduced expression of the affected β‐globin gene.^5^ Adult individuals heterozygous for Hb E usually have no clinical symptoms with Hb E ranging from 25% to 35% of the total hemoglobin.^6^, ^7^ In an individual with Hb E homozygote, the so‐called Hb EE disease, mild microcytic anemia can be observed. Without Hb A production, Hb E constitutes up to 80%‐90% of total hemoglobin.^8^ Evidence from previous studies in adults showed that the complex interactions of Hb E with α‐ and β‐thal could alter Hb E, F, and A_2_ levels,^9^, ^10^, ^11^ and this could result in difficulty making an accurate diagnosis. In young children, little information is available. In addition to thalassemia types, it is questionable for children under 24 months whether age and sex have a significant effect on the expression of these hemoglobins.

We used data from the “Lao Zinc study” a community‐based intervention trial among young Laotian children 6‐23 months of age at enrollment to evaluate the effect of age, sex, and different types of thalassemias on the expression of Hb F, A_2,_ and E in children with and without Hb E.

## 2 MATERIALS AND METHODS

### 2.1 Subjects and samples

The Lao Zinc Study was a randomized placebo‐controlled, double blind, community‐based trial implemented from September 2015 to April 2017 in Khammouane province, Lao PDR. The primary objective of the Lao Zinc study was to determine the effects of two forms of daily preventive zinc supplementation vs therapeutic zinc supplementation for diarrhea on young children's physical growth and other health outcomes.^12^ The study protocol and consenting procedure were approved by the National Ethics Committee for Health Research, Lao PDR; the Institutional Review Board of the University of California Davis, USA; and the Khon Kaen University, Thailand. The study procedures are described in detail elsewhere.^13^ Briefly, children were considered eligible if they were 6‐23 months of age, and their families accepted weekly home visits, planned to remain within the study area for the duration of the study and signed the informed consent document. Children were ineligible if they met one of the following criteria: severe anemia (Hb < 70 g/L), weight‐for‐length z‐score <−3 standard deviation,^14^ presence of bipedal edema, severe illness warranting hospital referral, congenital abnormalities potentially interfering with growth, chronic medical conditions (eg, malignancy) requiring frequent medical attention, known human immunodeficiency virus (HIV) infection of the index child or the child's mother, currently consuming zinc supplements, or currently participating in any other clinical trial. A total of 3433 infants and young children 6‐23 months of age were enrolled.

Laboratory diagnosis for thalassemia (including hemoglobin typing and DNA analysis) was done at baseline. For this purpose, a venous blood sample was collected into an evacuated 1.2‐mL polyethylene blood collection tube containing EDTA (Sarstedt AG & Co; ref 01.1604.400 and 06.1666.100, respectively). These samples were stored at 4‐8°C and transported to Thailand for analyses by an automated hemoglobin analyzer at Nakhon Phanom Hospital and polymerase chain reaction (PCR) techniques at the Center for Research and Development of Medical Diagnostic Laboratories (CMDL), Faculty of Associated Medical Science, Khon Kaen University in Thailand, as described in more detail below.

This add‐on study was approved by the Ethics Committees of Khon Kaen University (HE592006). All children who had complete laboratory analyses for Hb typing (n = 698) were recruited. Children were initially categorized into 3 main groups, that is, Group 1: Non‐Hb E (n = 272), Group 2: Heterozygous Hb E (n = 271), and Group 3: Homozygous Hb E (n = 155). Each group was subdivided further according to the number of α‐thal genes: no α‐thal gene, one α‐gene defect, and two α‐gene defects. One α‐gene defect included heterozygous states for α^+^‐thalassemia (α^+^‐thal), Hb Constant Spring (Hb CS), and Hb Pakse’ (Hb Ps’). All individuals with heterozygous α^0^‐thalassemia (α^0^‐thal), homozygous α^+^‐thal, homozygous Hb CS, and compound heterozygous states for α^+^‐thal/Hb CS (or Hb Ps’) were classified as having two α‐gene defects.

### 2.2 Laboratory methods for detection of thalassemia

All samples were investigated for Hb types and the corresponding fraction using the capillary zone electrophoresis (Capillarys II; Sebia, Lisses, France). Identification of α^0^‐thal mutations (SEA and THAI deletions) was performed using a multiplex PCR as described previously.^15^ Identification of deletional and nondeletional mutations causing α^+^‐thal, that is, 3.7 kb and 4.2 kb deletions, Hb CS and Hb Ps, was carried out using a multiplex gap‐PCR and allele specific PCR (ASPCR).^16^, ^17^ Additional analysis of β‐thal genes was carried out in cases suspected for β‐thal carrier, as indicated by Hb A_2_ > 3.5%.^18^, ^19^ Homozygosity for Hb E was tested in cases with either Hb EE or Hb EF phenotype using a previously described ASPCR.^9^

### 2.3 Statistical analysis

Data were tested for normality using the Shapiro‐Wilk test. Descriptive statistics, median, and interquartile range (IQR) were used to describe the levels of Hb F, A_2_, and E. Statistically significant differences among three or more independent groups were tested using the Kruskal‐Wallis test. The difference between two independent groups was compared with the Mann‐Whitney U test. Box plots were constructed to demonstrate the trend of change. To demonstrate the effect of age on Hb F, A_2_, and E levels, children were categorized according to age as 6‐12 months >12‐18 months, and >18‐23 months. Age group, sex, and thalassemia type were entered into the multiple regression model as independent variables. All graphics were constructed using the MedCalc Statistical Software version 18.11.6 (MedCalc Software bvba, Ostend, Belgium; https://www.medcalc.org; 2019). The STATA statistical software version 10.0 (StataCorp.) was used for multiple regression analysis. Statistical significance was set at *P < .05.*

## 3 RESULTS

### 3.1 Hb F, A_2,_ and E levels in children age 6‐23 months

**Figure 1:**
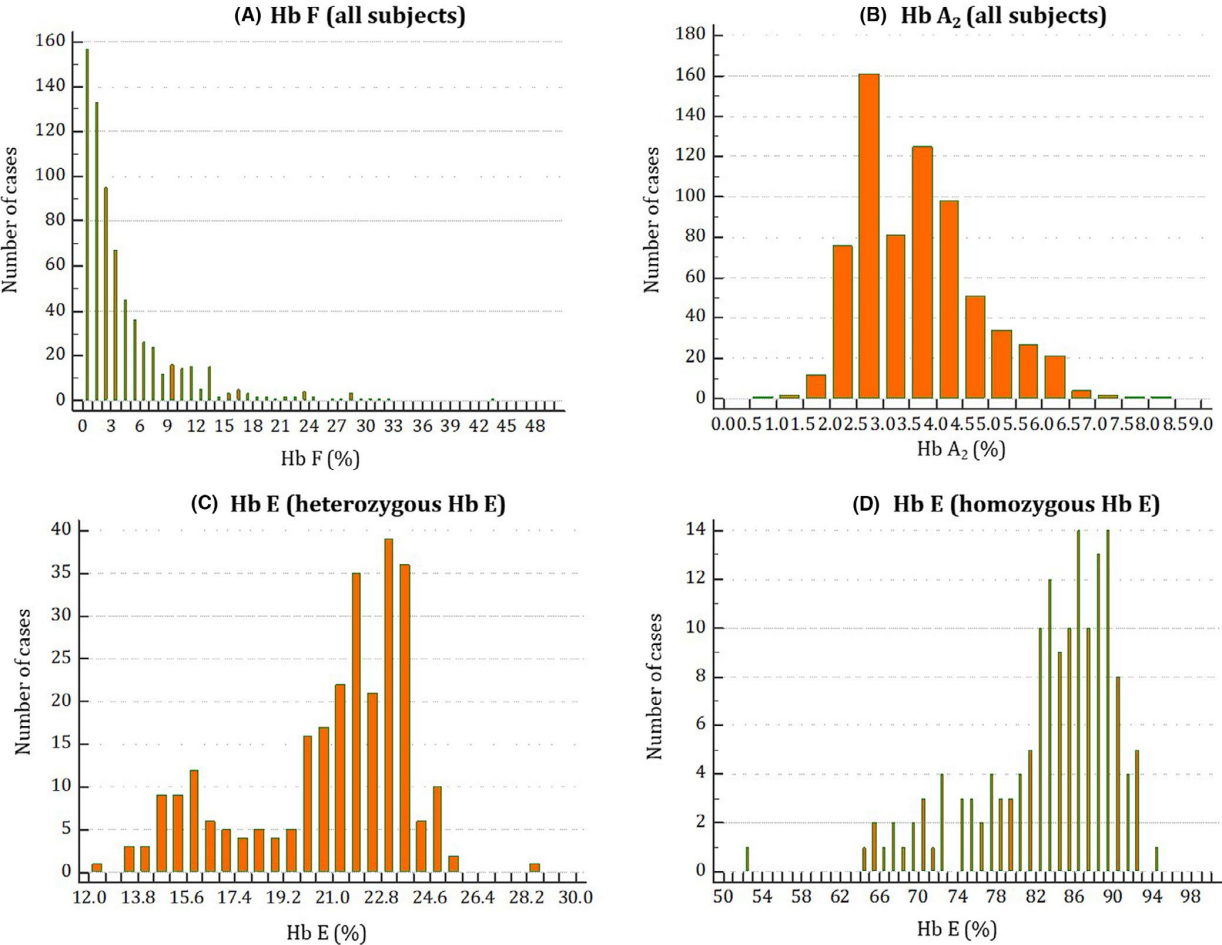
The distributions of Hb F, A_2,_ and E levels in Laotian children aged 6‐23 mo; (A): distribution of Hb F among total participants, (B): distribution of Hb A_2_ among total participants, (C): distribution of Hb E among Hb E heterozygotes, and (D): distribution of Hb E among Hb E homozygotes; X‐axis represents levels of Hb F, A_2,_ and E as indicated (expressed as % of total Hb); Y‐axis represents the number of cases [Colour figure can be viewed at wileyonlinelibrary.com]

The mean age of all children was 15.3 (SD = 5.2) months, and 368 (52.7%) were boys. The levels of each Hb in children aged 6‐23 months varied greatly, ranging from 0% to 43.7% for Hb F, 0.5 to 8.1 for Hb A_2_, 12.4%‐28.6% for heterozygous Hb E, and 52.9%‐94.1% for homozygous Hb E (Figure 1). The median values of Hb F, A_2,_ and E levels in children aged 6‐23 months, categorized by thalassemia types, are shown in Table 1.

**Table 1:** Hb F, A_2,_ and E levels in children aged 6‐23 mo, categorized by thalassemia types [data presented as median (IQR)]

Subject group	n	%Hb F^a^	%Hb A_2_ a	%Hb E^a^
Non‐Hb E	272	1.1 (0.5‐2.2)	2.6 (2.4‐2.8)	N/A
Without α‐thal gene (non‐thal)	129	1.1 (0.4‐2.1)	2.7 (2.4‐2.9)	N/A
One α‐globin gene defect^b^	95	1.0 (0.5‐2.0)	2.6 (2.4‐2.8)	N/A
Two α‐globin gene defects^c^	48	1.5 (0.7‐2.6)	2.2 (2.0‐2.5)	N/A
Heterozygous Hb E	271	2.7 (1.5‐4.5)*	3.8 (3.6‐4.1)*	21.9 (19.8‐23.1)
Without α‐thal gene	139	2.8 (1.4‐4.6)	3.8 (3.6‐4.1)	22.9 (21.8‐23.5)
With one α‐globin gene defect^b^	86	2.8 (1.6‐4.3)	3.8 (3.5‐4.1)	21.4 (20.3‐22.2)**
With two α‐globin gene defects^c^	46	2.6 (1.4‐4.7)**	3.9 (3.6‐4.2)	15.4 (14.8‐16.2)**
Homozygous Hb E	155	9.4 (6.0‐13.5)*	5.2 (4.5‐5.8)*	85.3 (80.9‐88.2)
Without α‐thal gene	65	10.6 (6.5‐20.4)	5.2 (4.3‐5.6)	83.4 (75.0‐87.3)
With one α‐globin gene defect^b^	64	8.3 (6.1‐13.0)	5.2 (4.5‐5.8)	86.2 (82.5‐88.4)***
With two α‐globin gene defects^c^	26	8.9 (5.0‐130)	5.4 (4.7‐6.0)	85.3 (82.5‐89.5)***

a Percent of total Hb.

b Including heterozygous states for α^+^‐thal, Hb CS, and Hb Ps.

c Including heterozygous states for α^0^‐thal, homozygous states for α^+^‐thal, Hb CS, and Hb Ps, and compound heterozygous states for α^+^‐thal/Hb CS.

* Significantly differed from non‐Hb E group (*P < .001*).

** Significantly differed from those without α‐thal gene (*P < .001*).

*** Significantly differed from those without α‐thal gene (*P = .017*).

Hb F levels in children heterozygous and homozygous for Hb E were significantly higher than that of non‐Hb E groups, that is, 2.7% (IQR = 1.5%‐4.5%) and 9.4% (IQR = 6.0%‐13.5%) vs 1.1% (IQR = 0.5%‐2.2%) (*P < .001*). Comparing within the non‐Hb E group, Hb F levels in non‐thal children and children with α‐thal genes did not differ significantly. Similarly, in children heterozygous and homozygous for Hb E, Hb F levels were not different between those without α‐thal genes and those with either one or two α‐gene defects (Figure S1).

Similar to Hb F, significantly higher Hb A_2_ levels were observed in children heterozygous and homozygous for Hb E, that is, 3.8% (IQR = 3.6%‐4.1%) and 5.2% (IQR = 4.5%‐5.8%) vs 2.6% (IQR = 2.4%‐2.8%) (*P < .001*). Among non‐Hb E children, a significantly reduced Hb A_2_ was seen in children with α‐thal (*P < .001*). However, in children heterozygous and homozygous for Hb E, the concomitance of α‐thal (either one or two α‐gene defect) did not result in significant changes in Hb A_2_ (Figure S2).

The median values of Hb E were 21.9% (IQR = 19.8%‐23.1%) for heterozygous Hb E and 85.3% (IQR = 80.9%‐88.2%) for homozygous Hb E. Among Hb E heterozygotes, Hb E levels reduced significantly in proportion to the number of α‐gene defects (*P < .001*). In contrary, children homozygous for Hb E coinherited with α‐thal (either one or two α‐gene defect) showed significantly higher Hb E levels, as compared with those without α‐thal (*P = .017) (*Supplementary Figure S3).

Subsequent analysis comparing the levels of Hb F and A_2_ between deletion and nondeletion α‐thal was done within a group of one α‐gene defect. Children with nondeletion showed significantly lower Hb A_2_ level, as compared with those with deletion (median value = 2.4% vs 2.6%; *P = .025*). For Hb F level, no significant difference was observed (Figure S4).

### 3.2 Effect of age, sex, and thalassemia types on the expression of Hb F, A_2_, and E

The results of multiple regression analysis are shown in Tables 2, 3, 4. Compared with children aged 6‐12 months, children aged >12‐18 months and >18‐23 months had a significant decline in Hb F expression (coefficient = −2.44 and −3.17). Females had 0.8% higher Hb F than males (*P = .006*). Similar to the finding in section 1 above, multiple regression analysis indicated that all forms of Hb E resulted in an increased Hb F levels with a coefficient range from 1.7 to 8.5 (Table 2).

**Table 2:** Results of multiple regression analysis demonstrating the effect of age, sex, and thalassemia gene on the expression of Hb F (Adjusted *R* ^2^ = .5394)

Factors	Coefficient	95% CI	*P* value
Age			
>12‐18 mo	−2.44	−3.15, −1.73	<.0001
>18‐23 mo	−3.17	−3.86, −2.48	<.0001
Sex			
Female	0.80	0.23, 1.37	.006
Thalassemia			
One α‐gene defect	−0.16	−1.16, 0.84	.754
Two α‐gene defect	0.63	−0.62, 1.89	.322
Heterozygous Hb E without α‐thal gene	2.13	1.23, 3.04	<.0001
Heterozygous Hb E with one α‐gene defect	1.71	0.68, 2.74	.001
Heterozygous Hb E with two α‐gene defects	1.74	0.47, 3.02	.007
Homozygous Hb E without α‐thal gene	12.2	11.02, 13.28	<.0001
Homozygous Hb E with one α‐gene defect	8.52	7.39, 9.66	<.0001
Homozygous Hb E with two α‐gene defects	7.23	5.63, 8.84	<.0001

**Table 3:** Results of multiple regression analysis demonstrating the effect of age, sex, and thalassemia gene, on the expression of Hb A_2_ (Adjusted *R* ^2^ = .7613)

Factors	Coefficient	95% CI	*P* value
Age			
>12‐18 mo	0.102	−0.003, 0.207	.056
>18‐23 mo	0.24	0.14, 0.34	<.0001
Sex			
Female	−0.12	−0.21, −0.04	.003
Thalassemia			
One α‐gene defect	−0.07	−0.21, 0.81	.374
Two α‐gene defect	−0.42	−0.62, −0.23	<.0001
Heterozygous Hb E without α‐thal gene	1.16	1.02, 1.29	<.0001
Heterozygous Hb E with one α‐gene defect	1.13	0.98, 1.28	<.0001
Heterozygous Hb E with two α‐gene defects	1.17	0.98, 1.36	<.0001
Homozygous Hb E without α‐thal gene	2.40	2.24, 2.57	<.0001
Homozygous Hb E with one α‐gene defect	2.45	2.28, 2.61	<.0001
Homozygous Hb E with two α‐gene defects	2.69	2.45, 2.92	<.0001

**Table 4:** Results of multiple regression analysis demonstrating the effect of age, sex, and the concomitance of α‐thal on the expression of Hb E in children heterozygous and homozygous for Hb E (Adjusted *R* ^2^ = .6680 for heterozygous Hb E and 0.2232 for homozygous Hb E)

Subject group	Factors	Coefficient	95% CI	*P* value
Heterozygous Hb E	**Age**			
	>12‐18 mo	0.18	−0.36, 0.72	.502
	>18‐23 mo	0.32	−0.21, 0.84	.240
	**Sex**			
	Female	0.12	−0.42, 0.44	.957
	**Concomitance of α‐thal**			
	With one α‐gene defect	−1.32	−1.79, −0.84	<.0001
	With two α‐gene defect	−6.96	−7.55, −6.36	<.0001
Homozygous Hb E	**Age**			
	>12‐18 mo	5.71	3.19, 8.22	<.0001
	>18‐23 mo	7.11	4.65, 9.57	<.0001
	**Sex**			
	Female	−0.62	−2.64, 1.41	.548
	**Concomitance of α‐thal**			
	With one α‐gene defect	3.31	1.11, 5.51	<.003
	With two α‐gene defect	5.92	2.98, 8.87	<.0001

There was a trend toward an increased Hb A_2_ expression with advancing age in which a statistical significance was attained in children age >18‐23 months (coefficient = 0.102 for age >12‐18 months and 0.24 for age >18‐23 months). Expression of Hb A_2_ was significantly lower in female than in male (coefficient = −0.12). Similar to that observed in the Hb F analysis, all forms of Hb E resulted in increased Hb A_2_ levels with a coefficient range from 1.13 to 2.69 (Table 3).

In Hb E heterozygotes, age and sex had no significant effect on Hb E levels (Table 4). However, a significantly increased Hb E with increasing age was observed in homozygous states (coefficient = 5.71 for age > 12−18 months and 7.11 for age >18−23 months). Based on a multiple regression analysis, the concomitance of α‐thal resulted in a reduction in Hb E of 1.32% for one α‐gene defect and 6.96% for two α‐gene defect, as compared with those without α‐thal gene (*P < .0001*). In contrast to Hb E heterozygotes, the coinheritance of α‐thal among Hb E homozygotes resulted in an increment of Hb E of 3.31% for a group with one α‐gene defect and 5.92% for a group with two α‐gene defects.

## 4 DISCUSSION

This study reports for the first time the levels of Hb F, A_2,_ and E in Laotian children aged 6‐23 months with and without Hb E. We found that the expression of these Hbs in the studied participants varied greatly, depending partly on thalassemia types. On average, Hb F and A_2_ levels of non‐Hb E children were similar to adult levels (ie, 1.1% for Hb F and 2.6% for Hb A_2_), suggesting that the Hb switching process in normal children and children with α‐thal may be completed within 2 years of age. In contrast, children heterozygous and homozygous for Hb E showed significantly higher Hb F and A_2_ levels than those of non‐Hb E groups. Considering the β^E^‐globin gene acts like β^+^‐thal,^5^ the higher Hb F and A_2_ levels are therefore explainable. With a shortfall of β^E^‐globin chain production, more α‐globin chains are available to combine with the δ‐ (for Hb A_2_) and γ‐globin (for Hb F) chains.^20^ The higher Hb F in children heterozygous and homozygous for Hb E may also indicate the delayed γ‐ to β‐globin switch. A similar result was observed in infants heterozygous for Hb E, and this was believed to be due to the β‐thalassemic effect of Hb E gene.^21^ However, the exact mechanism involved in this delay requires further in‐depth studies.

Applying multiple regression analysis, we found that in addition to thalassemia types, age and sex were associated significantly with the expression of Hb F and A_2_. In children aged >12‐18 months and >18‐23 months, Hb F was inversely related to age, indicating that age still plays a role in Hb F expression during this period of life. On the contrary to Hb F, a trend toward increased Hb A_2_ expression during 6‐23 months of age was observed though statistical significance was not reached during 12‐18 months of age. Similarly, results from an earlier study that followed the changes in Hb A_2_ levels in normal and β‐thal heterozygotes showed a gradually increased Hb A_2_ during the first year of life.^22^ These findings suggest that, despite the low expression, the δ‐globin gene may remain active during the first two years of life.

A few studies have reported the expression of Hb E during the neonatal period. It is evident in one study that the amounts of Hb E in infants heterozygous for Hb E increased sharply at 2‐3 months and reach its peak at 6 months of age.^21^ This is supported by our data that there was no association between age and Hb E level in Hb E heterozygotes. The increment of Hb E in heterozygous children aged >12‐18 months and >18‐23 months did not attain statistical significance, indicating that β^E^‐expression may reach its peak before 12 months. However, unlike Hb E heterozygotes, a significant association between age and Hb E level observed in children homozygous for Hb E. The fact that children homozygous for Hb E have only Hb E and Hb F; this significant association likely resulted from the reduced Hb F expression rather than increased Hb E production. As age increases, Hb F decreases; hence Hb E increases.

Similar to that observed in adults,^7^, ^10^, ^23^ the levels of Hb E were affected by the α‐thal coinheritance. A remarkable reduction was observed in children heterozygous for Hb E coinherited with two α‐gene defects. A previous study in infants also showed the same result.^21^ The explanation for this phenomenon relies on the electrostatic interaction of hemoglobin assembly. In Hb E heterozygotes, there are two types of β‐globin chains, the β^A^ and the more positively charged β^E^. When α‐globin is insufficiently produced, the α‐globin chain preferentially combines to β^A‐^ rather than β^E‐^chains. Hence, Hb E level is reduced in proportion to the number of α‐globin defects.

Unlike Hb E heterozygotes, a significant increment of Hb E was observed in children homozygous for Hb E coinherited with α‐thal (coefficient = 3.31 for one α‐gene defect and 5.92 for two α‐gene defects), and this was also the consequence of reduced Hb F. As observed in adults homozygous for Hb E,^8^, ^9^ the concomitance of α‐thal could result in a significant reduction in Hb F due to the preferential binding of α‐globin chain, which is limited, to β^E‐^globin rather than the γ‐globin chains.

Little information is known about the effect of sex on the expression of Hb F, A_2,_ and E. Previous studies reported that females had higher Hb F but lower Hb A_2_ levels, as compared with males.^24^, ^25^, ^26^ This is also the case for our study population. These data confirm that there might be some X‐linked factors involved in the expression of these two Hbs.

This study has limitation that iron deficiency (ID) was not excluded because of incomplete data on iron status. Whether ID has significant effect on the levels of Hb F, A_2,_ and E in young children requires further studies. Nonetheless, our study demonstrates a variation in Hb F, A_2_, and E expression in Laotian children 6‐23 months of age. The key factors contributing to the expression of Hb F and A_2_ appeared to be age and types of thalassemia, specifically Hb E and its interaction with α‐thal. Sex also had a significant effect on Hb F and A_2_ expression although the effect size was small. For Hb E, its expression appeared to rely mainly on the concomitance of α‐thal. It is of immense importance to note that a wide variation in Hb F levels could lead to the difficulty in thalassemia diagnosis at this particular age. This is especially true for children homozygous for Hb E as we found many cases with remarkably high Hb F levels. Without DNA analysis, these cases could be misdiagnosed as Hb E‐β‐thal disease. To differentially diagnose these two diseases, family study and DNA analysis are needed. Alternatively, the EE score recently described is probably useful for this differentiation.^27^, ^28^

## CONFLICT OF INTERESTS

The authors have no competing interests.
