# Supplementary figures and images for: Hemoglobins F, A2, and E levels in Laotian children aged 6‐23 months with Hb E disorders: Effect of age, sex, and thalassemia types
Source: Int J Lab Hematol. 2020 Feb 12;42(3):277–83. doi: 10.1111/ijlh.13164 (PMC7318314; doi:10.1111/ijlh.13164)

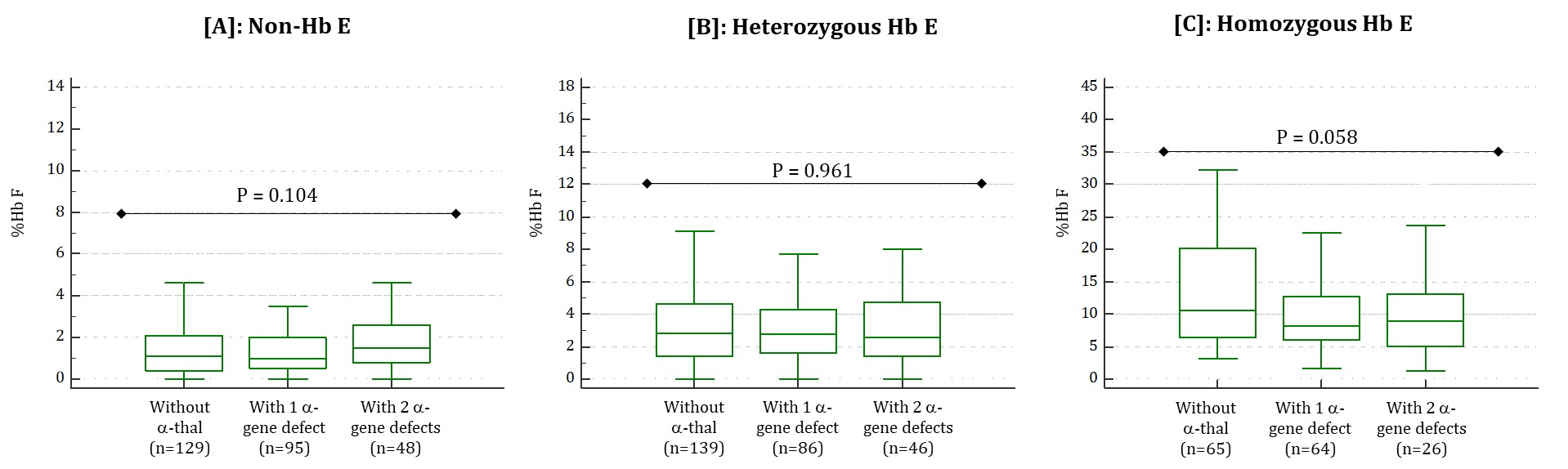

Supplement: Supplementary file 1 [file IJLH-42-277-s001.jpg]

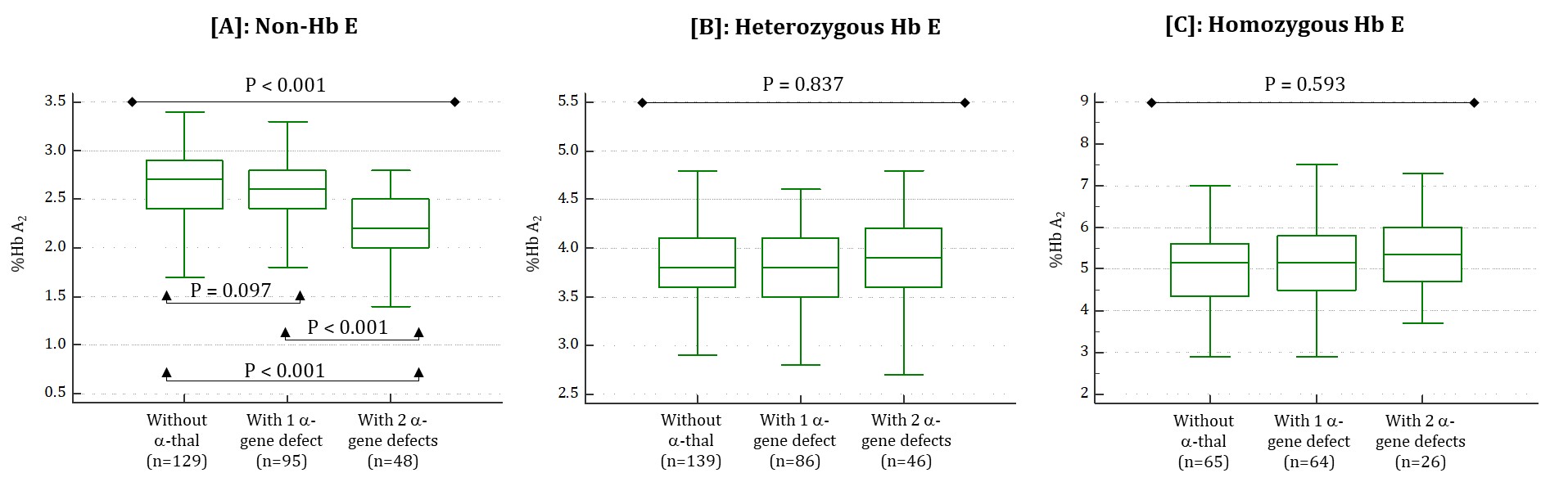

Supplement: Supplementary file 2 [file IJLH-42-277-s002.jpg]

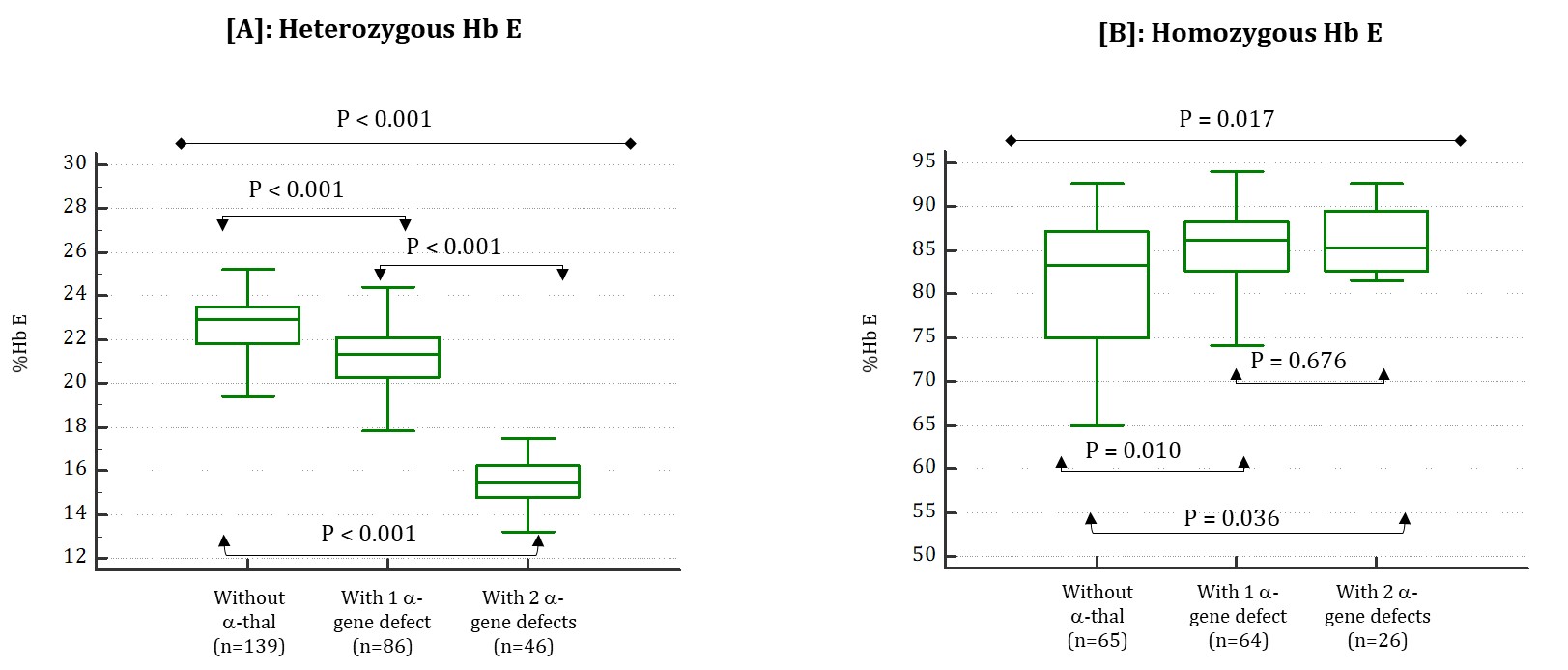

Supplement: Supplementary file 3 [file IJLH-42-277-s003.jpg]

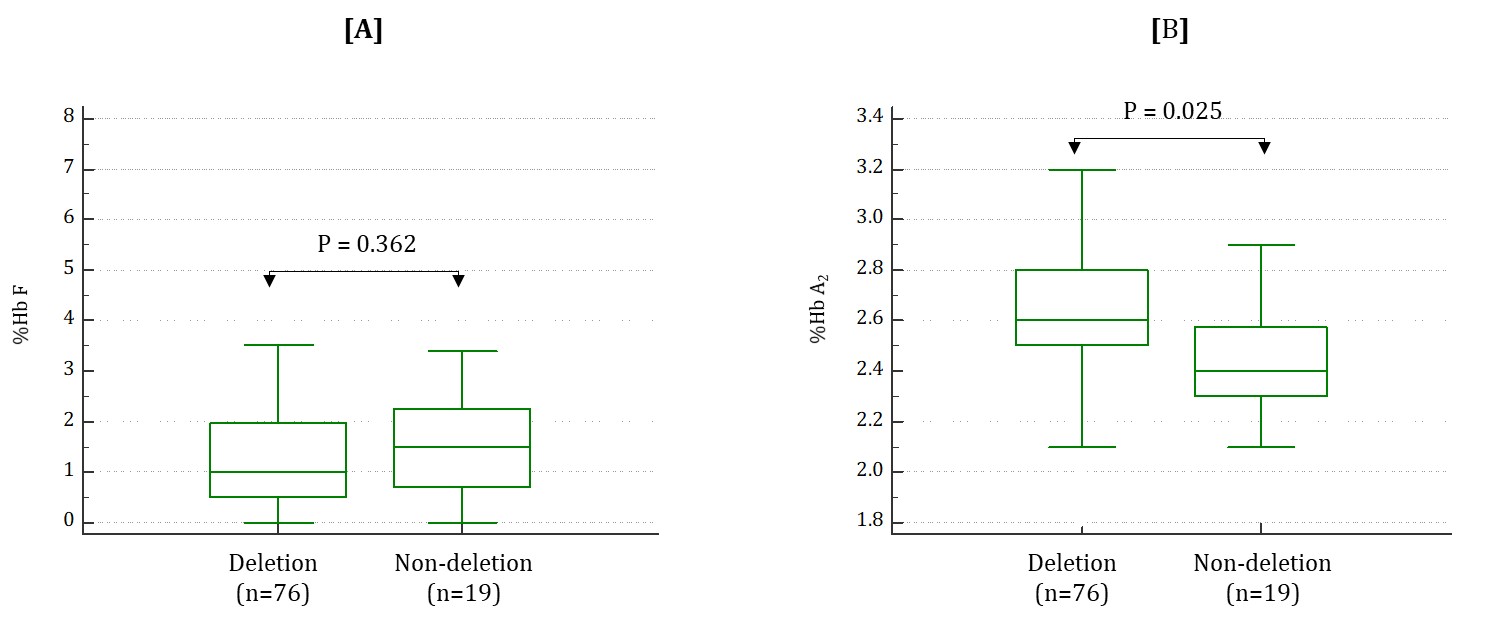

Supplement: Supplementary file 4 [file IJLH-42-277-s004.jpg]
